# Investigation of Epstein-Barr virus coinfection and changes in the gene expression of angiotensin converting enzyme II in peripheral blood mononuclear cells in COVID-19 patients

**DOI:** 10.1016/j.bbrep.2025.102311

**Published:** 2025-10-19

**Authors:** Hossein Lajmiri, Nioosha Ahmadi, Saeedeh Ebrahimi, Hadi Razavi Nikoo, Mehrdad Farrokhnia, Elham Heidari, Elham Mousavi

**Affiliations:** a Department of Medical Microbiology (Bacteriology and Virology), Afzalipour Faculty of Medicine, Kerman University of Medical Sciences, Kerman, Iran; b Medical Mycology and Bacteriology Research Center, Kerman University of Medical Sciences, Kerman, Iran; c Infectious Disease Research Center, Golestan University of Medical Sciences, Gorgan, Iran; d Department of Microbiology, Faculty of Medicine, Golestan University of Medical Sciences, Gorgan, Iran; e Infectious and Tropical Research Center, Kerman University of Medical Sciences, Kerman, Iran; f Department of Anatomy, School of Medicine, Iranshahr University of Medical Sciences, Iranshahr, Iran

**Keywords:** SARS-CoV-2, Epstein-Barr virus, Angiotensin-converting enzyme 2, Co-infection

## Abstract

SARS-CoV-2, a respiratory virus, causes a range of symptoms from mild cold-like signs to severe conditions like pneumonia and death. Co-infections, such as with Epstein-Barr Virus (EBV), may exacerbate disease severity. EBV can remain latent in B-cells and cause symptoms including pharyngitis, fatigue, and lymphopenia upon reactivation. Studies indicate that gene expression changes in the renin-angiotensin system, particularly involving angiotensin-converting enzyme 2 (ACE2), play a role in SARS-CoV-2 infection, and EBV might increase ACE2 expression. This study investigated EBV prevalence in plasma and peripheral blood mononuclear cells (PBMCs) of COVID-19 patients using serological (ELISA) and molecular (real-time PCR) methods, while also assessing ACE2 expression levels compared to healthy controls. Blood samples were processed to isolate PBMCs and plasma. Results revealed no IgM antibodies against EBV in plasma and only 2.18 % of PBMCs were EBV-positive, indicating no active EBV infection. ACE2 expression levels in PBMCs showed no significant difference between patients and controls. The study concludes that EBV reactivation is unlikely in these COVID-19 patients and recommends further investigation of ACE2 expression in nasopharyngeal epithelial cells.

## Highlights

• SARS-CoV-2 causes symptoms ranging from mild cold-like to severe pneumonia.
• Co-infections can increase COVID-19 severity and mortality risk.
• EBV may worsen COVID-19 symptoms like fatigue and lymphopenia.
• EBV might upregulate ACE2, potentially aiding viral infection.

## 1 Introduction

The onset of the Coronavirus disease 2019 (COVID-19), stemming from the severe acute respiratory syndrome coronavirus 2 (SARS-CoV-2), occurred in December 2019 [1]. Shortly after that, the swift proliferation of COVID‐19 globally compelled nations to acknowledge it as a pervasive global hazard [2]. Subsequently, this viral outbreak escalated into a worldwide pandemic, resulting in the loss of over 7.0 million lives [[3], [4], [5]]. SARS-CoV-2, akin to SARS-CoV, employs the cellular receptor angiotensin-converting enzyme II (ACE2) to facilitate entry into cells [6]. ACE2 is extensively present on the surfaces of the human airway and intestinal epithelial cells, making these cells highly prone to SARS-CoV-2 infection [7]. The virus is spread through droplets and aerosols [8]. The illness induced by these coronaviruses presents pneumonia-like symptoms such as fever and dry cough, eventually resulting in advancing respiratory impairment and potential fatality [9].

Coinfection cases involving SARS-CoV-2 and various bacterial, fungal, and respiratory viral pathogens have been documented with varying reported frequencies [[10], [11], [12]]. These coinfections among COVID-19 patients could potentially lead to elevated mortality rates [10,13]. Herpesviruses are recognized for their capacity to establish latent infections and reactivate in individuals with compromised immune systems [14]. Epstein-Barr virus (EBV), a double-stranded DNA virus from the herpesvirus family, is among the most prevalent viruses in the human population, as evidence of a prior infection is detected in over 90 % of adults globally [15]. The exact impact of SARS-CoV-2 on immune responses and its connection to the reactivation of opportunistic viral infections during the clinical progression of COVID-19 have not been definitively established [16]. However, multiple studies have reported occurrences where SARS-CoV-2 and EBV were concurrently detected [3,16,17]. Furthermore, evidence suggests that EBV can stimulate the expression of ACE2 and facilitate the entry of SARS-CoV-2 into epithelial cells [18].

Based on these findings, we chose to investigate the prevalence of EBV infection in the plasma and peripheral blood mononuclear cells (PBMCs) of COVID-19 patients using serology and molecular techniques. Subsequently, we evaluated the expression changes of ACE2 in patients compared to controls.

## 2 Materials and methods

### 2.1 Blood sample collection

Ninety-two whole blood specimens were obtained from diverse patients who received medical care at Afzalipour Hospital in Kerman, Iran, between February 10, 2022, and June 11, 2022, and then transferred into Complete Blood Count (CBC) tubes. Also, thirty healthy controls, frequency-matched by age and sex, were recruited from hospital staff and had no history of recent infection or chronic illness. All blood samples were collected upon admission. Additionally, all procedures and protocols for this study were approved by the ethical committee of Kerman University of Medical Sciences with national ethical approval #IR.KMU.AH.REC.1401.128. Specimens were collected during a period when the Omicron variant was prevalent in Iran.

### 2.2 PBMC isolation and plasma collection

Blood samples obtained from each patient's peripheral veins were collected, and PBMCs were isolated using Ficoll density-gradient centrifugation [19]. Approximately 5 mL of freshly collected peripheral blood samples in anticoagulant tubes was mixed with an equal volume of phosphate-buffered saline (PBS). The diluted samples were then carefully transferred into a 15 mL tube (BD Falcon) containing 5 mL of Ficoll-Paque Premium. After centrifugation for 20 min at 600 g, the PBMCs in the middle layer of the tubes were extracted and transferred to two new 1.5 mL microtubes for genome (DNA, RNA) extraction, and then washed twice with cold PBS. The supernatant was also separated as plasma into another microtube.

### 2.3 The enzyme-linked immunosorbent assay (ELISA)

Antibodies against VCA IgM were detected using ELISA kits from Vircell (Vircell Spain S.L.U., Granada, Spain), following the manufacturer's guidelines. Including positive and negative controls from the kit was crucial for ensuring the assay's reliability. Successful trials showed positive controls within an index absorbance range of 2–7 RLU and negative controls below 2 RLU. The cutoff value was determined by multiplying the average of the calibrators by the calibration factor. Cutoff values were obtained by dividing the sample absorbance values by their corresponding positivity index. Samples were classified as positive if their positivity index exceeded 1.10 RLU and negative if it was below 0.90 RLU.

### 2.4 Genome extraction

EBV DNA and total RNA from PBMCs were extracted using a kit (Blood Genomic DNA Extraction Mini Kit Favorgen) and Trizol reagent, respectively, following the manufacturers’ protocols.

### 2.5 Primer design

Three primers were designed by PISHGAM INDUSTRIAL EQUIPMENTS DESIGNERS COMPANY, including the Hypoxanthine-guanine phosphoribosyl transferase (HPRT) housekeeping gene, Epstein–Barr virus nuclear antigen 2 (EBNA-2), and ACE2 target genes. The primer sequences are shown in Table 1.

**Table 1:** Primer sequences.

Genes	Sequences
EBNA-2 (EBV)	F: 5′- GGGCATGGACCTCTAGCATC- 3′
	R: 5′- GGTCACCCCGTGATTGTCTTAC- 3′
ACE2 (Human)	F: 5′- CTGGGATCAGAGATCGGAAGAAG- 3′
	R: 5′- AGGAGGTCTGAACATCATCAGTG- 3′
HPRT(Human)	F: 5′- CCCTGGCGTCGTGATTAGTG- 3′
	R: 5′- TCGAGCAAGACGTTCAGTCC- 3′

### 2.6 cDNA preparation

To procure specific sequences of target genes, cDNA synthesis was carried out employing a kit from ParsTous Biotechnology following the manufacturer's instructions. Easy cDNA Synthesis kit contains all necessary components for conversion of total RNA or mRNA to single-stranded cDNA. The 2X Buffer mix solutions contain RT buffer, 1 mM dNTP mixture, 8 mM MgCl2, Oligo d(t)16, Random hexamer, and stabilizer. Enzyme mix contains thermostable H-minus MMLV, RNase Inhibitor, and stabilizer. In the process of cDNA synthesis, a mixture consisting of 10 μl of a 2X Buffer mix solution, 2 μl of reverse transcription enzyme buffer, and 8 μl of DEPC-treated water along with total RNA was combined. Subsequently, the PCR was executed using the following program: incubation for 10 min at 25 °C, followed by 60 min at 47 °C, and concluding with 5 min at 85 °C. Finally, the resulting PCR product was preserved at −70 °C. Following the completion of the cDNA synthesis, an optional quality assessment was performed to confirm the proper synthesis of the cDNA.

### 2.7 Real-time PCR assay

A total of 200 ng of cDNA and 2X Power SYBR® Green PCR Master Mix were utilized in the experimental setup. Each reaction mixture of 15 μL comprised 7.5 μL of 2X Master Mix, 1 μL each of forward and reverse primers, 2 μL of cDNA, and 4.5 μL of RNase-free water. The thermal cycling parameters on the Applied Biosystems Real-Time PCR system included an initial denaturation step at 95 °C for 15 min, followed by 40 cycles of denaturation at 95 °C for 30s, annealing at 54 °C for 30s, and extension at 72 °C for 30s, with a subsequent melting curve stage at 95 °C for 10 s and 72 °C for 1 min. The EBNA2 and ACE2 primer sets were employed for the real-time PCR-based identification of the EBV and ACE2 genes in the SARS-CoV-2 samples. Furthermore, HPRT primers were used as the human Internal Positive Control (IPC) in each sample. The final concentration of the primer mix was 10 pM. The threshold for positivity was Ct < 36. All samples were run in triplicate, with negative and positive controls included in each run.

### 2.8 Statistical analysis

All information presented in this investigation was articulated as Mean ± SD, as specified. To assess the equality of variances among the groups under comparison in parametric analyses, the F test was conducted. The statistical significance between two groups was examined using Student's t-test, while one-way analysis of variance (ANOVA) was utilized to identify significant differences across multiple groups. Analyses were performed in GraphPad Prism version 10 and SPSS 26, and results with P < 0.05 were deemed statistically significant.

## 3 Results

### 3.1 Demographics of COVID-19 patients

A total of 92 patients receiving care at Afzalipour Hospital in Kerman, Iran, between February and June 2022, were included in the study. The mean age of the participants was recorded at 64.32 ± 18.8 years. Among the study participants, randomly selected percentages were 54.3 % (n = 50) of non-ICU patients and 45.7 % (n = 42) of ICU-admitted patients with COVID-19 (Table 2).

**Table 2:** Demographics of study participants.

Demographic variables		COVID-19 (n = 92) N (%)	Controls (n = 30) N (%)
Age (y, mean ± SD)		64.32 ± 18.8	62 ± 12.3
Sex	Men	54 (58.7 %)	16 (53.3 %)
	Women	38 (41.3 %)	14 (46.7 %)
Ward	Non-ICU	50 (54.3 %)	–
	ICU-admitted	42 (45.7 %)	–
Mortality		34 (37 %)	–

### 3.2 ELISA

In adherence to the kit guidelines, an ELISA test was conducted on the patients' plasma samples. The qualitative outcomes of the assay were documented as either positive or negative. Based on the ELISA results, all samples tested negative for the presence of VCA IgM antibody specific to the EBV.

### 3.3 Frequency of the EBV genome in COVID-19 patients

The detection of viral genome presence was carried out through Real-time PCR analysis. Out of a total of 92 patients diagnosed with COVID-19, only a small percentage (2.18 %) showed positive results for the EBV genome. Both EBV DNA-positive patients were non-ICU cases; neither died during admission.

### 3.4 ACE2-mRNA expression in PBMCs of COVID-19 patients (ICU-admitted and non-ICU patients) compared with healthy controls

**Fig. 1:**
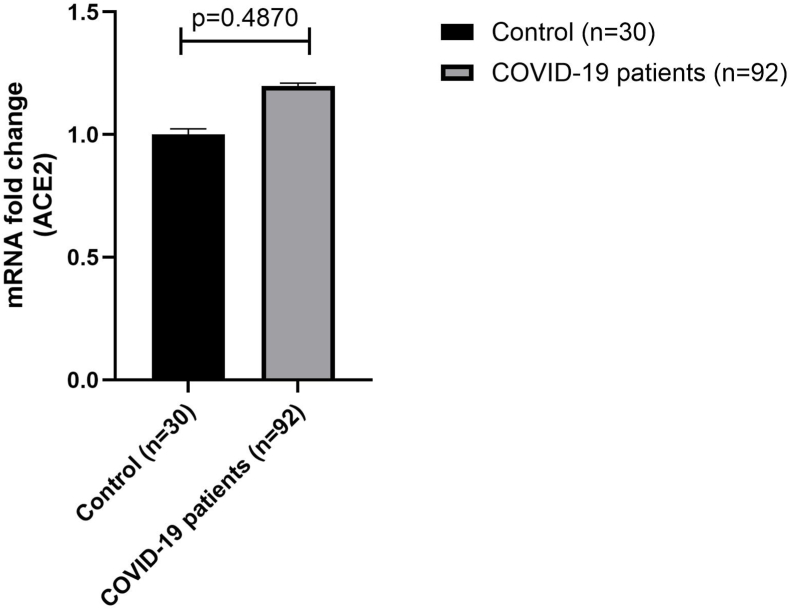
Comparison of ACE2 mRNA gene expression levels between healthy people (n = 30) and COVID-19 patients (n = 92) with error bars. ACE2 mRNA expression in PBMCs did not differ between COVID-19 patients (mean fold change: 1.19 ± 0.16, n = 92) and controls (1.00 ± 0.01, n = 30; *t*-test, p = 0.478).

**Fig. 2:**
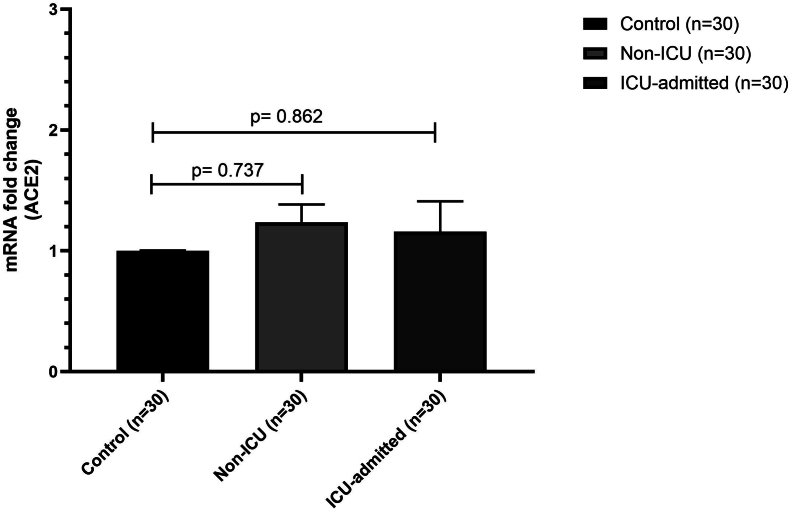
mRNA fold change of ACE2 in COVID-19 patients (ICU-admitted, n = 30, non-ICU, n = 30) compared with healthy controls (n = 30) with error bars. There was no significant (one-way ANOVA test) difference in the expression of ACE2 between ICU-admitted and non-ICU patients with healthy controls.

The expression levels of ACE2 mRNA were assessed in 2 different groups of PBMC specimens, such as COVID-19 patients and healthy people (without COVID-19). The expression level of ACE2 mRNA was calculated and contrasted with HPRT (housekeeping gene). Fig. 1 shows the mRNA fold change of ACE2 in patients compared with healthy people. Also, Fig. 2 indicates the ACE2 mRNA gene expression levels between all patients, _ICU-admitted and non-ICU patients_ with healthy people.

### 3.5 Comparison of ACE2 expression and EBV DNA status across clinical patient subgroups

Table 3 compares the presence of EBV DNA and levels of ACE2 gene expression across different patient subgroups, including ICU, Non-ICU, Deceased, Survivors, and based on EBV DNA status. Overall, ACE2 expression was higher in non-ICU patients, survivors, and those who were EBV DNA positive, but none of these differences were statistically significant (all p-values >0.05). This suggests that ACE2 expression does not significantly differ between these groups or in relation to EBV DNA status within this patient cohort.

**Table 3:** EBV DNA and ACE2 expression by clinical subgroups.

Subgroup	Patients n	EBV DNA + n	mRNA fold change of ACE2 (mean ± SD)	p-value
ICU-admitted	42	0	1.17 ± 1.05	0.192
non-ICU	50	2	1.65 ± 1.68	
Deceased	34	0	1.04 ± 0.85	0.124
Survivors	58	2	1.64 ± 1.62	
EBV DNA-positive	2	2	1.58 ± 0.08	0.158
EBV DNA-negative	90	0	1.22 ± 1.26	

## 4 Discussion

The SARS-CoV-2 pandemic has significantly changed healthcare systems worldwide. The objective of this study was to examine not only the direct effects of SARS-CoV-2 infection but also its interactions with Epstein–Barr virus (EBV) reactivation and the angiotensin-converting enzyme 2 (ACE2) receptor. By analyzing COVID-19 pathogenicity, EBV coinfection, and ACE2 expression levels, we offer a novel perspective on viral interactions during a global pandemic [20,21]. Since its emergence in late 2019, COVID-19 has been responsible for widespread morbidity and mortality [22].

The clinical manifestations of COVID-19 might range from a moderate upper respiratory tract disease to more serious conditions, including pneumonia and acute respiratory distress syndrome (ARDS) [23]. Many patients experience respiratory and gastrointestinal symptoms that are strongly linked to the virus's use of ACE2, a receptor widely expressed in airway and intestinal epithelial cells [24]. In our study, despite ACE2's importance for viral entry, quantitative PCR revealed no significant difference in ACE2 mRNA levels between COVID-19 patients and healthy controls. Also, no significant changes in ACE2 mRNA were observed in PBMCs from COVID-19 patients, regardless of ICU status, mortality, or EBV DNA positivity. This suggests that ACE2 expression in circulating immune cells does not correlate with disease severity or systemic viral load. These results align with the hypothesis that ACE2 regulation is tissue-specific; PBMCs may not reflect ACE2 modulation in primary SARS-CoV-2 target tissues (e.g., respiratory epithelium) [25]. Additional factors include sampling time (all specimens were collected at admission) and the potential influence of therapeutic agents. Serial measurements in respiratory or gastrointestinal samples are needed to clarify the dynamics of ACE2 expression.

A growing number of individuals are interested in how coinfections affect the course of COVID-19 [26]. One of these infectious agents that has attracted the public's interest is EBV, a common herpesvirus that may stay dormant and then reactivate when the immune system is weak [27]. EBV reactivation in the context of SARS-CoV-2 infection could potentially complicate the disease by further perturbing the immune response. We used both serological (ELISA for VCA IgM) and molecular (real-time PCR for EBV DNA) methods in our investigation to check for the presence of EBV. Our study found EBV DNA in PBMCs of only 2.18 % of COVID-19 patients, with no evidence of recent EBV infection by VCA IgM. This low rate is consistent with other reports [16] and may reflect the timing of sample collection, the transient nature of EBV reactivation, or technical detection limits. Although our findings did not indicate a significant prevalence of EBV, the few cases we identified should not be ignored, especially in individuals with severe COVID-19 who may be more likely to experience problems.

By concurrently assessing EBV status and ACE2 expression, we created a multidimensional framework for understanding COVID-19 pathogenesis. The rarity of EBV reactivation argues against it being a major driver of severe disease, yet isolated cases may signal underlying immunological vulnerability. In vitro data suggest EBV can upregulate ACE2 in epithelial cells, a hypothesis that merits investigation in respiratory tissue [18]. Our study didn't prove this relationship in the PBMC population, but it does establish the conditions for future research to find out if these kinds of interactions happen in other cell types or organs. This line of inquiry could be particularly relevant in understanding the pathophysiology of COVID-19 in patients with pre-existing EBV infections or in those who experience reactivation.

Our largely negative findings do not support a major role for EBV reactivation or PBMC ACE2 modulation in COVID-19 pathogenesis. However, rare EBV reactivation could still be clinically relevant in immunocompromised individuals and warrants further investigation.

This study has certain problems, even though it uses a new method. The sample size was not very big, and all of the patients came from the same hospital in Kerman, Iran. This might make it more difficult to use the results in larger groups of people with varied demographics or genetics. Additionally, limitations include cross-sectional design, lack of longitudinal follow-up, small number of EBV DNA-positive cases, and lack of direct analysis of respiratory tissues. Future studies should include multi-tissue, longitudinal sampling, and more granular clinical endpoints [2]. Furthermore, if future work confirms that EBV reactivation exacerbates COVID-19 in certain patients, antiviral or immunomodulatory strategies could be developed. Similarly, elucidating tissue-specific ACE2 regulation may inform therapies that modulate receptor expression or block viral entry, particularly in high-risk populations such as the elderly or those with comorbidities.

## 5 Conclusion

EBV reactivation is rare in hospitalized COVID-19 patients and does not correlate with ACE2 mRNA expression in PBMCs. Our results emphasize the need for larger, multi-tissue, and longitudinal studies to fully elucidate the interactions between latent viral infections and SARS-CoV-2.

## Data availability statement

The data that support the findings of this study are available on request from the corresponding author.

## Author's contributions

LH, AN, FM, KS-SM, and ME performed experiments, collected the data, and wrote the manuscript; ME is the supervisor. LH, R–NH, HE, ES, and ME analyzed the data, evaluated and interpreted the results; All authors approved the current version of the manuscript.

## Funding

There was no extramural funding received for conducting this study.

## Declaration of competing interest

The authors declare that they have no known competing financial interests or personal relationships that could have appeared to influence the work reported in this paper.

## Data Availability

The authors do not have permission to share data.
